# Content, Engagement, and Information Quality of Borderline Personality Disorder Videos on TikTok: Mixed Methods Analysis

**DOI:** 10.2196/106246

**Published:** 2026-10-16

**Authors:** Gabrielle Evoy, Simon Poirier, Majorie Vadnais, Alexandre Hudon

**Affiliations:** 1 Département de psychiatrie et d'addictologie, Faculté de médecine, Université de Montréal, 2900 Bd Édouard-Montpetit, Montréal, QC, H3T 1J4, Canada, 1 (514) 343-6111; 2 Département de psychiatrie, Programme des troubles relationnels et de la personnalité, Institut universitaire en santé mentale de Montréal, Montréal, QC, Canada; 3 Département de psychiatrie, Centre Hospitalier Universitaire Sainte-Justine, Montréal, QC, Canada; 4 Département de psychiatrie, Service de psychiatrie légale, Institut universitaire en santé mentale de Montréal, Montréal, QC, Canada; 5 Centre de recherche de l'Institut universitaire en santé mentale de Montréal, Montréal, QC, Canada; 6 Département de psychiatrie, Institut national de psychiatrie légale Philippe-Pinel, Montréal, QC, Canada; 7 Centre de recherche en pédagogie de la santé, Université de Montréal, Montréal, QC, Canada

**Keywords:** borderline personality disorder, personality disorders, TikTok, social media, mental health, health misinformation, qualitative research, thematic analysis, digital psychiatry, psychoeducation

## Abstract

**Background:**

TikTok has become a major source of mental health information, yet concerns persist regarding the quality and reliability of psychiatric content disseminated on the platform. While misinformation has been documented for several mental disorders, no study has specifically examined how borderline personality disorder (BPD) is portrayed on TikTok.

**Objective:**

This study aims to characterize TikTok content related to BPD by describing its thematic content, engagement patterns, presenter characteristics, and indicators of information quality using a mixed methods secondary analysis.

**Methods:**

This mixed methods observational study performed a secondary analysis of a previously established dataset of publicly available TikTok videos related to mental health. A total of 207 videos addressing BPD or BPD-like traits were included. Quantitative analyses summarized video characteristics, engagement metrics, presenter backgrounds, content type, intent, and authenticity indicators using descriptive statistics. Qualitative data were analyzed using reflexive thematic analysis following Braun and Clarke’s methodology to identify recurrent narratives related to BPD.

**Results:**

Among the 207 included videos, 185 (89.4%) were in English and 199 (96.1%) targeted the general public. Most creators (n=124, 59.9%) did not disclose professional credentials, whereas psychiatrists (n=18, 8.7%) and psychologists (n=16, 7.7%) represented the largest identified professional groups. Seven overarching themes were identified: understanding personality disorders, treatment and management, interpersonal relationships, myths and misconceptions, co-occurring disorders, personal experiences, and diagnosis and classification. Videos focused on personal experiences generated the highest audience engagement (mean weighted likes 2389, SD 6529; favorites 333, SD 707; shares 117, SD 287), whereas treatment-focused videos demonstrated substantially lower engagement (mean weighted likes 222, SD 360; favorites 54, SD 77; shares 19, SD 40). Opinion-based or mixed-content formats predominated across most themes. Reference-based information remained limited, ranging from 0% to 33.3% depending on thematic category, while misinformation was observed across multiple themes, particularly understanding personality disorders (20/81, 24.7%) and diagnosis and classification (14/48, 29.2%). Experiential and relational narratives were substantially more prominent and engaging than evidence-based psychoeducational content.

**Conclusions:**

TikTok discourse surrounding BPD is dominated by experiential narratives that foster engagement but frequently rely on opinion-based rather than evidence-based information. Although many videos may provide validation and reduce stigma, the limited use of references and the persistence of misinformation highlight the need for the greater involvement of mental health professionals in producing accessible, accurate, and engaging digital content. To our knowledge, these findings provide the first focused overview of BPD-related content on TikTok and may inform future strategies to improve the quality of online mental health information.

## Introduction

Personality disorders are a pervasive pattern of maladaptive traits and behaviors beginning in adolescence or early adult life, leading to substantial personal distress or social dysfunction, or both, and disruption to others [1]. The most currently used classification (*Diagnostic and Statistical Manual of Mental Disorders, Fifth Edition, Text Revision* [*DSM-5-TR*]) describes 10 distinct personality disorders, the best-known one being borderline personality disorder (BPD), which affects up to 5.9% of the American population [2,3]. Defining characteristics of BPD are instability in interpersonal relationships, identity, impulsivity, and affect. To make a diagnosis according to the *DSM-5*, 5 out of 9 diagnostic criteria need to be met, the most predictive ones for a correct diagnosis being suicidal tendencies and unstable relationships [4]. BPD has an important impact on the quality of life and functioning of affected individuals but can be successfully addressed with proper care, based on psychotherapy [4]. Specific therapies for BPD, such as dialectical behavioral therapy (DBT) or mentalization-based therapy, tend to have a long-term approach and remain scarcely available in most countries, which represents a barrier to accessing specialized care [4,5]. Moreover, the culture of stigma and lack of education in the general and medical population regarding personality disorders limit help-seeking in affected people [5]. This context might encourage people to look for answers elsewhere, notably on social media.

The use of social media has been increasing over the last decades, with TikTok quickly becoming one of the most popular platforms; over 1.5 billion people used TikTok monthly as of 2025 to find a wide array of content tailored to their interests, including information about mental health [6]. Indeed, as much as 70% of American adults consult the internet in search of health information [7]. At the time of writing, there were 23.9 million posts tagged with #mentalhealth on TikTok, 66,500 with #personalitydisorder, 464,900 with #borderlinepersonalitydisorder, and 2.1 million with #BPD [8]. At this scale, it can be challenging to distinguish videos that provide reliable information from those that spread misinformation. Misinformation is defined as false or misleading information accidentally disseminated without malicious intent [9]. It differs from disinformation, which involves the intentional dissemination of false or misleading information with the objective of misleading or influencing the public [10].

Health care misinformation on social media is a well-documented phenomenon that has mostly been studied in the context of infectious diseases and vaccines [11]. Recent studies also report that content related to mental health on TikTok is of poor quality in many cases. Aragon-Guevara et al [12] found that in the 133 most popular videos tagged with #autism, 41% presented inaccurate information and 32% were potentially misleading when compared to clinical guidelines. The findings of Yeung et al [13] suggest that about half of attention-deficit/hyperactivity disorder (ADHD)–related content found on TikTok is misleading, especially when posted by non–health care providers. This is important because false information online may lead to mistrust of medical professionals and institutions, as well as treatment reluctance, which can in turn delay adequate diagnosis and care [14]. In the field of mental health, the spread of misinformation and disinformation may also exacerbate loneliness and stigma in an already vulnerable population [15]. While social media has the potential to be an educative tool with impressive reach, it is crucial that health care professionals use critical thinking skills and media literacy to help their patients navigate the vast amount of online content and help them find reliable information [16]. To our knowledge, no study has yet been conducted to analyze the quality of content related to personality disorders on TikTok.

The aim of this study is to explore the content found on social media regarding BPD. We wish to describe and understand the content that is shared on TikTok, assess which type of content drives the most viewer engagement, and determine which, if any, characteristics are most strongly associated with disinformation. Another objective is to draw general recommendations for medical professionals when sharing content online, with regard to the specific challenges related to personality disorders.

## Methods

### Study Design

This study used a secondary mixed methods observational design, drawing on an existing dataset of TikTok videos collected as part of a larger international investigation into mental health–related disinformation on social media [17]. The present project focused specifically on content related to BPD and combined quantitative descriptive analyses with an in-depth qualitative thematic analysis. The qualitative component followed a reflexive thematic analysis approach and constituted the primary analytic contribution of this project. Given the observational design of this study, reporting followed the STROBE (Strengthening the Reporting of Observational Studies in Epidemiology) guidelines.

### Data Source, Subset Selection, and Sample Characteristics

The dataset originated from a previously published observational study that analyzed 1000 publicly accessible TikTok videos addressing a wide range of mental health topics across English, French, and Spanish. That parent study applied a structured framework for social-media information quality, adapted from the taxonomy proposed by previous work and described in detail in the original study, to characterize content, perceived intent, and authenticity [17]. Statistical and machine learning approaches were subsequently used in the parent study to examine predictors of misleading information [17].

The videos in the parent dataset were published between September 2016 and June 2024, providing a cross-sectional corpus spanning several years of TikTok’s development and changes in online mental health discourse [17]. In the parent study, candidate videos were initially identified by monitoring popular mental health–related hashtags and search trends, including #ADHD, #mentalhealth, #therapy, #anxiety, #depression, and #mentalhealthawareness, and the sample was subsequently broadened using TikTok’s algorithmically curated *For You* feed. Newly created neutral accounts without prior engagement histories were used across sampling sessions to reduce personalization bias. Eligible videos addressed a mental health topic; contained or were intended to contain psychoeducational material; were available in English, French, or Spanish; were self-contained rather than part of a multipart series; and were publicly accessible without requiring a TikTok account. No restrictions were imposed according to creator type, while spam or bot accounts and extremely short videos without identifiable mental health content were excluded [17].

Each video in the parent dataset was manually classified according to its main mental health topic by trained members of the research team, with each classification counter-validated by a second rater. For the present secondary analysis, we extracted all 207 videos classified under the parent dataset’s broader “personality disorders” category. All 207 videos specifically addressed BPD. Thus, the present study represents a BPD-specific secondary analysis rather than an analysis of personality disorders more broadly.

### Quantitative Data and Variables

Quantitative variables were drawn directly from the parent dataset and were used to describe the characteristics of the selected videos. These variables included video duration, language, country of origin when available, engagement indicators normalized by time since posting, presenter self-identified professional status, and intended target audience. Indicators related to information quality, including content type, perceived intent, and authenticity markers, were also retained. In the present study, these quantitative elements were not used to generate predictive models but rather to contextualize and enrich interpretation of the qualitative findings, particularly in relation to the prominence, tone, and reach of specific narratives about BPD. To account for differences in the amount of time videos had been available on the platform, engagement metrics were normalized according to time since upload. For each video, weighted likes, comments, favorites, and shares were calculated by dividing the cumulative number of the respective interaction by the number of days elapsed since the video was uploaded. Thus, weighted engagement metrics represent the average number of interactions per day.

The content, intent, and authenticity variables used in the present secondary analysis were coded during the construction of the parent dataset and were not reassessed for the present study [17]. The coding framework was adapted from previous work and characterized 3 dimensions of information quality. Content was classified as opinion-based, fact-based, or mixed. For perceived intent, disinformation denoted false information or claims unsupported by the scientific community, misinformation denoted information that was only partially consistent with current scientific knowledge, clickbait referred to content primarily intended to promote a product or service, satire used humor or parody to convey information, and “other” encompassed informative, educational, or experiential content not meeting these criteria. Authenticity referred to the apparent basis or source of the information: framing indicated selective, decontextualized, or interpretively presented information, whereas reference-based content explicitly mentioned or cited an identifiable external source. Factual accuracy was evaluated by trained coders according to consistency with current scientific knowledge and consensus. The parent coding procedure did not involve a formal item-by-item comparison with a prespecified set of clinical guidelines; rather, classifications reflected trained researcher judgments that were counter-validated by a second coder and resolved by consensus when discrepancies occurred [17].

In the parent study, 8 members of the research team received standardized training in the application of the coding framework. Each video was coded and subsequently counter-validated by another member of the research team, such that all videos were assessed by at least 2 researchers. Before full coding, a pilot exercise was conducted on a random sample of 50 videos to calibrate interpretation and resolve discrepancies. Interrater reliability was evaluated for categorical coding decisions, with overall agreement of 87%; disagreements were resolved through discussion and consensus [17]. These ratings should be distinguished from the reflexive thematic analysis conducted specifically for the present study, which was performed collaboratively by GE and AH as described below.

### Quantitative Analysis

Descriptive statistics were computed to summarize the distribution of these variables and to identify dominant trends within the BPD-related content, without the aim of hypothesis testing or predictive modeling. Quantitative results were used interpretively rather than inferentially, serving to situate thematic patterns in relation to visibility, engagement, and content characteristics on TikTok. All quantitative analyses were performed using standard descriptive procedures, and the findings were integrated narratively with the qualitative results to support a mixed methods interpretation of how BPD is portrayed and disseminated on social media. This approach is consistent with the analytic framework and data structure of the parent study while reflecting the exploratory and interpretive focus of the present secondary analysis.

Quantitative analyses were conducted using Python 3.14 (Python Software Foundation) with the scikit-learn suite.

### Qualitative Analytic Framework

A reflexive thematic analysis was conducted following the methodological principles outlined by Braun and Clarke [18]. This approach was selected to allow for an interpretive examination of both explicit and implicit meanings embedded in video content. The analysis sought to capture not only what was stated about BPD but also how the disorder was framed; who was positioned as an authority; how lived experience was represented; and how responsibility, stigma, and recovery were implicitly constructed. Both manifest content, such as explicit claims about diagnosis or treatment, and latent content, such as underlying assumptions about identity or pathology, were considered throughout the analytic process.

### Qualitative Data Analysis

All qualitative analyses were conducted collaboratively by GE and AH, both of whom possess clinical and research expertise in psychiatry and personality disorders. The analytic process began with repeated familiarization with the video content to develop an in-depth understanding of recurring narratives and discursive patterns. Initial codes were generated inductively and refined iteratively through regular analytic discussions. Rather than aiming for interrater reliability in a positivist sense, reflexive dialogue was used to challenge assumptions, clarify interpretations, and enhance conceptual depth. Discrepancies in interpretation were resolved through discussion and return to the data, ensuring that themes were grounded in the content while remaining analytically coherent.

Qualitative coding and thematic analyses were conducted using QDAMiner (Provalis Research). Individual videos could generate multiple initial codes during the reflexive thematic analysis. However, for the purposes of descriptive quantitative comparison across thematic categories, each video was assigned to 1 primary overarching theme. The primary theme was determined according to the dominant narrative or principal focus of the video. When content plausibly encompassed more than 1 theme, GE and AH reviewed the video collaboratively and reached consensus regarding the theme that best represented its primary focus. Accordingly, 7 primary thematic categories were mutually exclusive at the video level and together accounted for all 207 videos.

### Ethical Considerations

This study did not require formal ethics approval, as it involved secondary analysis of publicly available content and did not include interaction with individuals or access to private or identifiable information. This determination was consistent with the ethical assessment conducted for the parent study and aligned with the Canadian Tri-Council Policy Statement on Ethical Conduct for Research Involving Humans [19]. All data have been deidentified.

To further protect creator privacy and reduce the possibility of reverse identification through online searches, illustrative examples presented in the *Results* section were paraphrased rather than reproduced verbatim. These paraphrases were designed to preserve the substantive meaning and thematic content of the original videos while removing distinctive wording. Usernames, account handles, profile information, URLs, video identifiers, and other direct identifiers were not reported.

## Results

### Video Characteristics

Among the TikTok videos addressing personality disorders included in this secondary analysis, the majority did not specify a country of origin. The video characteristics are presented in Table 1. Specifically, 70.1% (n=145) of the videos lacked identifiable geographic attribution, reflecting the frequent absence of contextual metadata on TikTok. When a country was specified, the United States accounted for the largest proportion of videos (n=35, 16.9%), followed by the United Kingdom (n=12, 5.8%), Australia (n=12, 3.3%), Canada (n=5, 2.4%), and France (n=3, 1.4%). Given the high proportion of videos without identifiable geographic information, no firm conclusions can be drawn regarding geographic representation. However, the sample was predominantly English-language, indicating a clear linguistic predominance of English-language content.

**Table 1.** Sociodemographic and descriptive characteristics of TikTok videos addressing borderline personality disorder (N=207).

Category and characteristic	Values, n (%)
Country	
Not specified	145 (70.0)
United States	35 (16.9)
United Kingdom	12 (5.8)
Australia	7 (3.3)
Canada	5 (2.4)
France	3 (1.4)
Total	207 (100)
Presented by (professional title)	
Not specified	124 (59.9)
Psychiatrist	18 (8.7)
Psychologist	16 (7.7)
Therapist	11 (5.3)
Coach	10 (4.8)
MA psychology, PhD candidate	8 (3.8)
Social worker	4 (1.9)
Brainspotting practitioner	2 (0.9)
Certified clinical trauma specialist and narcissistic abuse recovery professional	1 (0.4)
Comedian	1 (0.4)
Counselor	1 (0.4)
Doctor	1 (0.4)
Influencer	1 (0.4)
Licensed resident in counseling	1 (0.4)
Medical doctor	1 (0.4)
Medical resident	1 (0.4)
Medical student	1 (0.4)
Mental health worker and advocate	1 (0.4)
Neuropsychologist	1 (0.4)
Neuroscientist	1 (0.4)
Podcaster	1 (0.4)
Somatic hypnotherapist	1 (0.4)
Total	207 (100)
Language	
English	185 (89.3)
French	20 (9.6)
Spanish	2 (0.9)
Total	207 (100)
Target audience	
General public	199 (96.1)
Specific audience	8 (3.8)
Total	207 (100)

Most videos were presented by creators who did not disclose a professional title or credentials, representing nearly 60% (n=124) of the sample. Among videos with identifiable professional backgrounds, psychiatrists (n=18, 8.7%) and psychologists (n=16, 7.7%) were the most frequently represented, followed by therapists (n=11, 5.3%) and coaches (n=10, 4.8%). A smaller proportion of videos were presented by individuals with academic training in psychology, such as master’s-level clinicians or doctoral candidates. A wide range of other presenter identities was observed, including social workers, medical trainees, neuroscientists, and mental health advocates, each accounting for less than 2% (n=1-4) of the sample. A small number of videos were produced by non–mental health professionals, including a comedian and a podcaster, underscoring the heterogeneity of voices contributing to online discourse about BPD.

The linguistic distribution of videos was highly skewed toward English, which accounted for 89.4% (n=185) of the sample. French-language content represented 9.7% of the videos (n=20), while Spanish-language videos were rare, comprising less than 1% (n=2) of the dataset. This linguistic imbalance mirrors broader trends observed on TikTok and aligns with the geographic distribution of identified countries.

With respect to intended audience, the overwhelming majority of videos were directed toward the general public. Only 3.9% (n=8) of the videos explicitly targeted a specific audience, such as individuals with a diagnosis of BPD, caregivers, or clinicians. This predominance of general-audience framing suggests that most BPD-related content on TikTok is designed for broad consumption rather than tailored psychoeducation or professional discourse.

### Thematic Analysis Results

#### Overview

The thematic analysis of the 207 TikTok videos addressing BPD revealed a heterogeneous yet recurrent set of narratives that collectively shape how BPD is understood, experienced, and evaluated on social media. Content oscillated between psychoeducational explanations, experiential testimony, and value-laden interpretations of behavior and relationships. Seven overarching themes were identified, capturing both informative and problematic dimensions of online discourse: conceptualizations of personality disorders, treatment and management, interpersonal relationships, myths and misconceptions, co-occurring disorders, personal experiences, and diagnostic framing. Together, these themes illustrate how TikTok functions simultaneously as a space for mental health education, identity construction, peer validation, and, at times, simplification or stigmatization of complex psychiatric phenomena. A summary of the themes and their characteristics is presented in Table 2.

**Table 2.** Summary of themes identified in TikTok videos addressing borderline personality disorder.

Theme	Core focus	Description of content	Illustrative examples of narratives
Understanding personality disorders	Conceptualization of BPD^a^	Videos defining BPD and describing its core characteristics, including emotional dysregulation, fear of abandonment, identity instability, and impulsivity. Content ranged from clinically aligned explanations to simplified symptom lists aimed at broad audiences	Explanations of “what BPD is,” emotional intensity metaphors, lists of common signs and traits
Treatment and management	Care and coping strategies	Discussion of evidence-based treatments, such as dialectical behavior therapy, emotion regulation skills, and recovery trajectories, alongside barriers to accessing specialized care. Medication was variably framed as supportive or ineffective	DBT^b^ skill explanations, accounts of long waitlists, advice on self-management
Interpersonal relationships	Relational impact of BPD	Narratives focusing on romantic, familial, and social relationships, emphasizing attachment insecurity, fear of abandonment, and relational conflict. Some content targeted loved ones, while other videos centered lived relational experiences	Push-pull dynamics, advice for partners, explanations of attachment patterns
Myths and misconceptions	Stigma and misinformation	Explicit attempts to counter stigmatizing beliefs about manipulation, attention-seeking, or emotional volatility, alongside occasional reinforcement of stereotypes through oversimplified framing	Reframing “manipulation” as fear-based behavior, challenging moral judgments
Co-occurring disorders	Psychiatric comorbidity	Exploration of BPD in conjunction with other mental health conditions such as anxiety disorders, depression, substance use disorders, and trauma-related conditions, often highlighting diagnostic overlap and complexity	Discussions of misdiagnosis, overlap with bipolar disorder or ADHD^c^
Personal experiences	Lived experience and identity	First-person narratives describing emotional suffering, recovery, stigma, and self-understanding. Videos often served peer-support and validation functions and emphasized authenticity and community	Testimonies of daily life with BPD, recovery journeys, advice to peers
Diagnosis and classification	Diagnostic framing	Content addressing diagnostic criteria, subtypes such as “quiet BPD,” and comparisons with other personality or mood disorders. Diagnosis was portrayed as both validating and stigmatizing	Self-identification vs clinical diagnosis, subtype explanations

a BPD: borderline personality disorder.

b DBT: dialectical behavior therapy.

c ADHD: attention-deficit/hyperactivity disorder.

#### Theme 1: Understanding Personality Disorders

This theme encompassed videos aiming to define BPD and describe its core characteristics, symptomatology, and emotional patterns. Content frequently framed BPD through accessible language, emphasizing emotional intensity, fear of abandonment, identity instability, and affective dysregulation. While some videos aligned closely with established clinical descriptions, others relied on simplified or dramatized portrayals, often using relatable scenarios or brief symptom checklists to foster recognition and engagement.

Examples of content included short explanatory segments distinguishing BPD from mood disorders, visual lists of “common signs,” and metaphor-based explanations of emotional reactivity. These videos often positioned themselves as educational introductions rather than comprehensive clinical accounts.

Illustrative paraphrased examples of verbatims are as follows:

*BPD was described as involving unusually intense emotional experiences rather than simply exaggerated or dramatic behavior.*

*Individuals with BPD were portrayed as experiencing certain emotions, particularly fear of abandonment and rejection, with heightened intensity.*

#### Theme 2: Treatment and Management

Videos grouped under this theme addressed therapeutic approaches, coping strategies, and structural barriers to care. Dialectical behavior therapy was frequently cited as the gold standard, often presented alongside practical skills such as distress tolerance or emotion regulation. Medication was discussed more variably, sometimes framed as adjunctive support and other times portrayed skeptically or dismissed altogether.

A recurrent narrative concerned difficulties accessing specialized treatment, long waitlists, and feelings of being misunderstood or dismissed by health care systems. Some content emphasized self-help strategies and peer-based coping in the absence of formal care, reflecting both resilience and systemic gaps.

Illustrative paraphrased examples of verbatims are as follows:

*Some creators described DBT as highly beneficial, while also emphasizing substantial delays in accessing treatment.*

*Medication was portrayed as having a limited role in treating BPD itself, whereas coping and emotion-regulation skills were described as more helpful for managing intense emotional states.*

#### Theme 3: Interpersonal Relationships

This theme focused on the relational impact of BPD, particularly in romantic and familial contexts. Videos frequently described cycles of idealization and devaluation, intense attachment needs, fear of abandonment, and relational conflict. Some content was directed at partners or loved ones, offering explanations for behaviors perceived as confusing or overwhelming.

While certain videos adopted an empathetic tone, emphasizing mutual understanding and communication, others framed individuals with BPD as inherently “difficult” in relationships, reinforcing polarized narratives of victimhood and blame.

Illustrative paraphrased examples of verbatims are as follows:

*Relationships involving someone with BPD were described as sometimes alternating between intense closeness and interpersonal distancing.*

*Relational difficulties were often framed as stemming from fears of abandonment rather than from a deliberate wish to damage the relationship.*

#### Theme 4: Myths and Misconceptions

This theme explicitly addressed stigma, challenging common misconceptions surrounding manipulation, attention-seeking, and emotional volatility. Many creators attempted to reframe behaviors often labeled as “manipulative” as maladaptive coping responses rooted in fear, trauma, or emotional dysregulation.

At the same time, some videos inadvertently reinforced stereotypes by using provocative language or absolutist claims. The coexistence of stigma-challenging and stigma-reinforcing content highlighted the ambivalence of social media discourse around BPD.

Illustrative paraphrased examples of verbatims are as follows:

*Behaviors sometimes interpreted as manipulative were instead framed as responses to intense fears of abandonment.*

*Rapid shifts in emotional states were described as genuine experiences rather than evidence that the emotions were insincere.*

#### Theme 5: Co-Occurring Disorders

Many videos situated BPD within a broader psychiatric landscape, discussing frequent comorbidities such as substance use disorders, anxiety, depression, posttraumatic stress disorder, and eating disorders. Content often emphasized diagnostic overlap and the risk of misdiagnosis, particularly confusion with bipolar disorder or ADHD.

These narratives underscored the complexity of clinical presentation and the challenges of disentangling overlapping symptoms, sometimes advocating for more nuanced diagnostic assessments.

Illustrative paraphrased examples of verbatims are as follows:

*Substance use was described as a common co-occurring difficulty in BPD, sometimes framed as a way of coping with overwhelming emotional distress.*

*BPD was portrayed as potentially occurring alongside anxiety and trauma-related difficulties rather than as an isolated condition.*

#### Theme 6: Personal Experiences

First-person narratives constituted a substantial portion of the dataset and were often framed as testimony, validation, or peer support. These videos described lived experiences of emotional pain, identity confusion, self-harm urges, recovery journeys, and the struggle for self-compassion. Many creators used storytelling to counter stigma and foster a sense of community.

Advice directed at peers and loved ones was common, with an emphasis on boundaries, patience, and self-awareness. Emotional authenticity was a defining feature of this theme.

Illustrative paraphrased examples of verbatims are as follows:

*Living with BPD was described as involving persistent internal conflict and psychological distress.*

*Recovery was framed not as eliminating intense emotions, but as developing ways to understand and manage them more effectively.*

#### Theme 7: Diagnosis and Classification

This theme addressed diagnostic criteria, assessment processes, and subtypes of BPD, including references to constructs such as “quiet BPD.” Videos often contrasted BPD with other personality disorders or mood disorders, sometimes accurately and sometimes simplistically.

Diagnostic labeling was portrayed ambivalently: for some, diagnosis was validating and empowering; for others, it was associated with stigma, fear, or clinical rejection. Content occasionally blurred the line between formal diagnosis and self-identification.

Illustrative paraphrased examples of verbatims are as follows:

*Receiving a BPD diagnosis was described as providing an explanatory framework for previously confusing experiences.*

*“Quiet BPD” was portrayed as involving substantial internal distress despite symptoms being less outwardly visible.*

### Video Characteristics by Thematic Category

Video characteristics varied widely across thematic categories, reflecting differences in narrative focus, audience engagement, and content depth. Videos centered on understanding personality disorders represented the largest thematic group and were generally concise, with an average duration of 1.68 (SD 1.49) minutes and moderate engagement levels. Content within this theme attracted an average of 959 (SD 2227) weighted likes and relatively low comment activity, suggesting broad visibility but limited interactive discussion. Diagnosis and classification videos were slightly longer on average and generated higher comment volumes, indicating greater audience engagement around diagnostic labeling, subtype distinctions, and self-identification processes. Interpersonal relationships constituted a smaller but highly engaging category, with longer average video durations and elevated interaction metrics, particularly favorites and shares, highlighting the resonance of relational narratives with viewers. Personal experience videos, although fewer in number, generated the highest engagement across most metrics, including likes, favorites, and shares, underscoring the strong affective and identificatory pull of first-person accounts. In contrast, treatment and management videos were the longest on average but showed the lowest observed engagement across several metrics. Videos addressing myths and misconceptions demonstrated moderate engagement despite their brevity, while co-occurring disorders represented a small subset with relatively modest engagement metrics. Collectively, these patterns indicate that relational and experiential content related to BPD was associated with higher observed engagement than treatment-focused or corrective psychoeducational material. Video characteristics by thematic category are presented in Table 3.

**Table 3.** Characteristics and engagement metrics by thematic category in TikTok videos addressing borderline personality disorder.

Topic	Number of videos	Video length (min), mean (SD)	Weighted likes, mean (SD)	Weighted comments, mean (SD)	Weighted favorites, mean (SD)	Weighted shares, mean (SD)
Understanding personality disorders	81	1.68 (1.49)	959 (2227)	20 (38)	184 (378)	82 (179)
Diagnosis and classification	48	1.72 (1.49)	1082 (6000)	66 (393)	144 (610)	99 (557)
Interpersonal relationships	34	2.13 (1.54)	1483 (3541)	29 (57)	372 (878)	129 (253)
Personal experience	18	1.96 (1.68)	2389 (6529)	22 (37)	333 (707)	117 (287)
Treatment and management	14	2.54 (2.41)	222 (360)	6 (7)	54 (77)	19 (40)
Myths and misconceptions	8	1.27 (0.88)	622 (917)	14 (25)	147 (247)	43 (75)
Co-occurring disorders	4	1.57 (1.15)	417 (505)	13 (14)	109 (125)	82 (88)

### Content Characteristics, Intent, and Authenticity by Thematic Category

Marked differences were observed across thematic categories in terms of content type, perceived intent, and authenticity indicators. These are presented in Table 4. Videos focused on understanding personality disorders predominantly relied on mixed content combining opinion and factual elements, while purely opinion-based and fact-based content were equally represented. Within this theme, misinformation was more common than disinformation, and a substantial proportion of videos were classified as having an intent categorized as “other,” reflecting broadly educational or experiential aims. Authenticity markers frequently fell under framing or non–reference-based sources, with fewer than 1 in 5 videos explicitly citing references.

**Table 4.** Content type, perceived intent, and authenticity indicators by thematic category in TikTok videos addressing borderline personality disorder.

Topic	Number of videos	Content (%): opinion-based, fact-based, mixed	Intent (%): disinformation, misinformation, clickbait, satire, other	Authenticity (%): rumor, propaganda, hoax, conspiracy, framing, reference-based, other
Understanding personality disorders	81	27.16, 27.16, 45.68	9.88, 24.69, 3.70, 1.23, 60.49	7.41, 0.00, 0.00, 0.00, 24.69, 19.75, 48.15
Diagnosis and classification	48	12.50, 56.25, 31.25	4.17, 29.17, 4.17, 2.08, 60.42	4.17, 0.00, 0.00, 0.00, 12.50, 33.33, 50.00
Interpersonal relationships	34	55.88, 5.88, 38.24	8.82, 29.41, 0.00, 2.94, 58.82	17.65, 0.00, 0.00, 0.00, 38.24, 2.94, 41.18
Personal experience	18	72.22, 5.56, 22.22	0.00, 16.67, 0.00, 11.11, 72.22	5.56, 0.00, 0.00, 0.00, 27.78, 5.56, 61.11
Treatment and management	14	42.86, 21.43, 35.71	7.14, 14.29, 7.14, 0.00, 71.43	14.29, 7.14, 0.00, 0.00, 0.00, 28.57, 50.00
Myths and misconceptions	8	25.00, 25.00, 50.00	12.50, 12.50, 0.00, 25.00, 50.00	12.50, 0.00, 0.00, 0.00, 37.50, 12.50, 37.50
Co-occurring disorders	4	50.00, 0.00, 50.00	50.00, 0.00, 25.00, 0.00, 25.00	25.00, 0.00, 0.00, 25.00, 0.00, 0.00, 50.00

Diagnosis and classification videos differed notably, with over half presenting fact-based content and a higher proportion of reference-based authenticity, suggesting greater alignment with formal diagnostic discourse despite persistent levels of misinformation.

In contrast, interpersonal relationships and personal experience themes were overwhelmingly opinion-based, with minimal fact-based content. These videos were largely categorized under nondeceptive intent and relied heavily on framing or personal narrative rather than external references, highlighting their experiential rather than informational orientation. Treatment and management content showed a more balanced distribution between opinion-based and mixed content, with relatively low levels of misinformation and moderate use of reference-based sources, though engagement with formal evidence remained inconsistent. Videos addressing myths and misconceptions displayed substantial heterogeneity, including the highest relative proportion of satire, reflecting attempts to counter stigma through humor or provocative framing. Finally, content related to co-occurring disorders, although scarce, showed the highest relative proportion of disinformation and clickbait intent, alongside limited use of references.

Overall, these patterns indicate that themes grounded in lived experience and relational narratives are more likely to privilege opinion-based and framed content, whereas diagnostic and classification-focused videos more frequently incorporate factual and reference-based elements, albeit alongside persistent misinformation.

## Discussion

### Summary of the Main Findings

To our knowledge, this study provides the first focused mixed methods examination of BPD-related content on TikTok. Across the videos, content was largely directed toward a general audience, predominantly English-language, and frequently presented by creators without disclosed professional credentials. Thematic analysis revealed 7 recurring narrative domains spanning psychoeducation, lived experience, relational dynamics, diagnostic framing, treatment, comorbidity, and stigma-related misconceptions. Quantitative patterns indicated that experiential and relational themes generated the highest engagement, while treatment-focused and corrective content showed comparatively limited reach. Moreover, substantial variability was observed in content quality, intent, and authenticity across themes, with opinion-based and framed narratives predominating and reference-based information remaining relatively uncommon.

### Comparison With Prior Works

Importantly, the thematic content identified in this study was not uniformly inconsistent with established clinical knowledge about BPD. Several themes showed substantial concordance with the contemporary BPD literature. Videos grouped under understanding personality disorders frequently emphasized emotional dysregulation, fear of abandonment, identity instability, impulsivity, and interpersonal difficulties, all of which correspond closely with the core clinical features of BPD [20,21]. Similarly, treatment and management content commonly emphasized psychotherapy and particularly dialectical behavior therapy (DBT), while expressing greater skepticism regarding medication. This broadly reflects current evidence: psychotherapy remains the treatment of choice for BPD, with several structured approaches, including DBT, mentalization-based treatment, transference-focused psychotherapy, and schema therapy, demonstrating efficacy, although no single approach has consistently demonstrated superiority [21]. Likewise, pharmacotherapy has not demonstrated consistent efficacy for the core features of BPD and is generally considered adjunctive, targeted toward specific symptoms or comorbid disorders rather than the disorder as a whole [21,22]. Thus, the informal or experiential presentation of information on TikTok should not necessarily be interpreted as evidence of clinical inaccuracy.

Other themes similarly reflected well-established dimensions of BPD while also illustrating how clinical phenomena can be reframed through social-media discourse. Interpersonal dysfunction is a defining feature of BPD and can produce substantial distress within romantic and family relationships; systematic reviews have documented emotional burden, role strain, and difficulties experienced by significant others of individuals with BPD [23]. Likewise, portrayals of people with BPD as “manipulative,” “attention-seeking,” or intentionally difficult have a longstanding history in clinical settings and contribute to the particularly high degree of stigma associated with the diagnosis [24]. The frequent discussion of co-occurring depression, anxiety, trauma-related disorders, substance use, and eating disorders also parallels the high psychiatric comorbidity consistently documented among individuals with BPD [21,25]. In contrast, diagnostic and classification content demonstrated a more evident tension between social-media terminology and formal psychiatric nosology. Constructs such as “quiet BPD,” although used online to describe individuals whose symptoms are perceived as more internalized, are not recognized as distinct BPD subtypes in *DSM-5-TR*. Their prominence illustrates how social media may generate informal diagnostic categories that facilitate self-recognition and community identification while potentially blurring the boundaries between clinically established diagnostic constructs and popular mental health terminology.

The prevalence of opinion-based and mixed content observed in this study aligns with prior research documenting variable quality of mental health information on TikTok and other social media platforms. Studies examining autism- and ADHD-related content have similarly reported high rates of misleading or incomplete information, particularly when content is produced by non–health care professionals [12,13]. Our findings extend this literature to personality disorders, a domain characterized by greater diagnostic complexity and stigma. Consistent with broader misinformation research, content framed as experiential or educational in intent was more common than overt disinformation, yet misinformation remained prevalent across several themes [9]. This distinction is clinically relevant, as even nonmalicious inaccuracies may shape illness perceptions, self-diagnosis, and treatment expectations. Prior work has shown that repeated exposure to misleading health information can erode trust in clinicians and institutions, ultimately delaying engagement with evidence-based care [14].

The high engagement observed for themes centered on personal experience and interpersonal relationships mirrors findings from digital mental health research emphasizing the affective and identificatory power of first-person narratives. Social media platforms privilege emotionally resonant content, and storytelling has been shown to foster perceived authenticity, validation, and community belonging among users [16,20]. In the context of BPD, such narratives may serve adaptive functions by countering stigma and normalizing symptoms and their associated distress. However, they may also contribute to diagnostic oversimplification or identity consolidation around illness labels, particularly when narratives blur the line between lived experience and diagnostic criteria. This tension has been noted in prior qualitative analyses of mental health discourse online, where self-identification may precede or replace formal assessment, raising concerns about misdiagnosis and unmet clinical needs [9].

The prominence of themes related to stigma, myths, and barriers to treatment access is consistent with longstanding literature on BPD. BPD remains one of the most stigmatized psychiatric diagnoses, both in the general population and within health care settings, contributing to reluctance to seek care and difficulties accessing specialized psychotherapy [4,5]. Our findings suggest that BPD-related content on TikTok may simultaneously challenge and reinforce stigma, depending on framing and intent. While some creators explicitly reframed behaviors labeled as manipulative as fear-driven coping responses, others reproduced reductive or moralizing narratives. The relatively low engagement observed for treatment and management content indicates that, within this sample, evidence-based information such as psychotherapy principles or system-level barriers generated fewer interactions than several experiential and relational forms of content. This pattern underscores the need for clinicians to engage thoughtfully with social media, balancing accessibility and emotional resonance with clinical accuracy [16]. It is also important for medical practitioners to be able to address these issues with patients in a sensitive manner, while bringing a critical lens to the discussion.

The temporal context of the sampled content should also be considered. Videos in the underlying dataset were published between September 2016 and June 2024, a period during which public discourse surrounding BPD may have been influenced by highly visible media events. One notable example is the 2022 Depp-Heard defamation trial, during which expert testimony concerning BPD received extensive social-media attention. Fang and Zhu [26] found that the trial was associated with increased stigmatizing and decreased destigmatizing language concerning personality disorders on X (formerly Twitter). Such events may temporarily alter the visibility, framing, and language surrounding psychiatric diagnoses across social-media platforms. However, because the present study did not examine temporal trends or stratify content according to upload date, we cannot determine whether the trial or other contemporaneous events influenced the themes or information-quality patterns observed in our sample. Future longitudinal studies could examine how online representations of BPD change in response to major media events.

### Limitations

Several limitations should be considered when interpreting these findings. This study relied on a secondary analysis of publicly available TikTok videos, which limits insight into creator intent and audience interpretation. Content categorization, particularly regarding intent and authenticity, involved interpretive judgment, although analytic reflexivity was used to enhance rigor. The sample was predominantly English-language and drawn from a single platform, restricting generalizability to other cultural or digital contexts. Engagement metrics reflect platform dynamics rather than clinical impact and cannot be equated with informational quality or therapeutic value. Finally, the cross-sectional nature of the dataset precludes conclusions regarding longitudinal effects of exposure to specific content types on attitudes, help-seeking behaviors, or clinical outcomes. Further work could focus on qualitative interpretation of patients’ lived experience while seeking mental health information online, providing direct insight into the perspective of social media users.

### Conclusions

BPD-related content on TikTok is characterized by a strong emphasis on lived experience, interpersonal relationships, and diagnostic interpretation, with these forms of content attracting greater engagement than treatment-focused information. Much of the discourse reflects clinically recognizable aspects of BPD, while misinformation, limited referencing, and informal diagnostic constructs remain important concerns. These findings highlight the potential dual role of TikTok as a source of validation and community as well as a potential source of oversimplification and inaccurate mental health information. Whether similar patterns occur on other social-media platforms requires further study. Mental health professionals should therefore engage with social-media content critically while recognizing the importance of the experiences and concerns that resonate with online audiences.

## Abbreviations

ADHD: attention-deficit/hyperactivity disorder
BPD: borderline personality disorder
DBT: dialectical behavior therapy
*DSM-5*: *Diagnostic and Statistical Manual of Mental Disorders, Fifth Edition*
*DSM-5-TR*: *Diagnostic and Statistical Manual of Mental Disorders, Fifth Edition, Text Revision*
STROBE: Strengthening the Reporting of Observational Studies in Epidemiology
